# An Undergraduate Curriculum in Public Health Benchmarked to the Needs of the Workforce

**DOI:** 10.3389/fpubh.2015.00012

**Published:** 2015-02-17

**Authors:** J. Michael Stoots, Randy Wykoff, Amal Khoury, Robert Pack

**Affiliations:** ^1^Department of Community and Behavioral Health, College of Public Health, East Tennessee State University, Johnson City, TN, USA; ^2^Department of Health Services Management and Policy, College of Public Health, East Tennessee State University, Johnson City, TN, USA

**Keywords:** undergraduate public health, workforce, bachelor of public health, public health job market, benchmarking

East Tennessee State University (ETSU) has offered an undergraduate degree in public health for 60 years. Alumni survey data have documented that the majority of the graduates from this program enter the workforce [see accompanying commentary by Wykoff, et al. (1)]. To keep pace with ongoing changes in the workforce, the decision was made to completely review, and, as appropriate, revise and restructure the Bachelor of Science in Public Health (BSPH) curriculum.

While the specific curricular revisions were adopted to address the recognized workforce needs of the region of central Appalachia where ETSU is located, the process undertaken, and the resulting curricular outcomes could be useful models for other undergraduate programs where the majority of graduates enter the workforce upon graduation.

Consistent with the College’s strategic plan, a BSPH Re-structuring Taskforce was formed including three department chairs, the academic dean, a student representative, and the BSPH coordinator (Stoots). The taskforce reviewed data from a variety of systematically collected assessments, including the annual alumni surveys, the bi-annual employer surveys, and the field preceptor evaluations, which are completed at the end of each student’s mandatory internship. The taskforce also reviewed the exit survey, conducted at the time of student graduation, as well as data from the students’ culminating presentations, both of which ask students to specifically comment on ways in which the program could be improved. Throughout the process, the taskforce interfaced with faculty, staff, students, and employers.

To facilitate the interviews with the preceptors and employers, the taskforce utilized a framework that emphasized six questions:

(1) What knowledge and skills will (your profession) require in 5 years?
(2) What *issues* should all (your profession) graduates be able to *discuss*?
(3) What *things* should all (your profession) graduates be able to *do*?
(4) What *tools* should all (your profession) graduates be able to *use*?
(5) What *problems* should all (your profession) graduates be able to *solve*?
(6) What *characteristics* should all (your professions) graduates be able to *exemplify*?

Once all recommendations from the various sources were gathered, the taskforce conducted a comprehensive qualitative analysis using the card-sorting technique. Through their analysis they identified potential competency domains and approximately 400 desired learning objectives. (This list is posted at: http://www.etsu.edu/cph/academics/undergraduate/bspublichealth.aspx).

A group of faculty, staff, and students was then assembled as the Undergraduate Curriculum Workgroup and was chaired by the BSPH Coordinator. This group worked to map the identified competency domains and learning objectives, in an “introduced” and “reinforced” format, into the coursework. This format reduced redundancy by only having a concept introduced once, and then mapping all concept reinforcements so they would build upon each other.

While this process began prior to the release of the ASPH Recommended Critical Component Elements for an Undergraduate Major in Public Health^1^, the competency domains were subsequently mapped against the Critical Component Elements, and found to be congruent and comprehensive.

In addition to the identification of the competency domains and the learning objectives, four over-arching themes emerged from this process:

(1) Employers seek graduates who are knowledgeable in their field, but who also possess cross-cutting skills related to professional and ethical behavior;
(2) Employers value graduates who have very strong written and verbal communication skills;
(3) Employers expect graduates to have expanded technological capabilities, particularly with Microsoft applications (e.g., Excel) and electronic health records; and
(4) Students want more exposure to working professionals in the field, prior to their internship.

The first two themes were addressed by incorporating specific skills and projects related to professionalism and communication skills into each core course. The third theme was addressed by enhancing the technology-requirements of the core courses and by revising and expanding the existing “Emerging Technologies for the Health Professions” course. This course now incorporates a greater focus on software usage, spreadsheet tools, and electronic presentation in order to prepare students for the newly added technology components in the core courses.

The final theme was addressed in several ways:

(1) First, a one-credit hour “Skills and Encounters” course was added to each of the four semesters prior to the semester-long internship. The “Skills and Encounters” courses will expose students to a range of public health workforce settings and introduce a cross-section of skills essential for workplace success, including “professionalism,” “career preparation,” and “teamwork,” among others. For example, throughout the various Skills and Encounters courses, in addition to direct on-site visits with working professionals, students will engage in a variety of scenarios with progressively challenging responsibilities related to conduct in work settings, job interviews, and professional communication.
(2) Second, students will complete the ESSENTIALS course, a hands-on/applied course that teaches students to make a range of products required for improving health in low-resource settings (e.g., water filters, composting latrines, adobe structures). By presenting students with a range of problems that they have never faced – from using new tools to constructing items without all of the necessary supplies – ESSENTIALS requires students to work in teams, to think creatively, and to solve an array of logistical and operational challenges, while, at the same time, developing an appreciation for the realities associated with living in resource-poor environments.

Table 1 shows the BSPH curriculum after the revision, and includes the BSPH core courses as well as the Community Health and Health Care Administration concentrations.

**Table 1:** **Undergraduate public health curriculum benchmarked to the needs of the workforce**.

**TABLE CURRICULUM: AFTER REVISION**
**Public health core (46 credit hours)**	**Community health concentration (15)**
Biostatistics (3 credit hours)	Cultural competencies and spirituality in health care (3)
Emerging technologies for the health professions (3)	Community organization for health education programs (3)
Principles of epidemiology (3)	Behavior change theory for public health (3)
Health services administration (3)	Service grant writing (3)
Health systems (3)	Lifespan health promotion (3)
Principles and practices of public health education (3)	**Health care administration concentration (15)**
Environmental sanitation (3)	Legal and ethical issues in healthcare (3)
First aid and emergency care (3)	Health services planning (3)
Public health budgeting and finance (3)	Quality and utilization assurance (3)
Top 5 health threats (3)	Current issues in health services management and policy I (1)
Skills and encounters I (1)	Current issues in health services management and policy II (2)
Skills and encounters II (1)	Health informatics (3)
Skills and encounters III (1)	
Skills and encounters IV (1)	
Essentials (3)	**Minor: 18 credits (required for both concentrations)**
Field experience (9)	

The BSPH revision represents three distinct and fundamental changes. The first is the methodology used to drive the curricular revision. The processes involved all stakeholders – employers, students, preceptors, and faculty – at every juncture. Second, it was explicitly designed to prepare students for the workforce, with significantly increased focus on practical/workforce experiences, skills, and experiential learning for the students. The third outcome is the nature of the change in the curriculum – with a greater focus on professionalism, communication skills, technological, and cultural competence. These changes reflect a commitment to assuring that graduates are prepared, as well as possible, for entry into the local health workforce.

While the specific curricular outcomes may vary in different parts of the country, we believe that this systematic, workforce-oriented, approach, should be relevant for many undergraduate public health programs, especially those where the majority of students enter the workforce upon graduations.

## Conflict of Interest Statement

The authors declare that the research was conducted in the absence of any commercial or financial relationships that could be construed as a potential conflict of interest.

## Footnotes

^1^http://www.aspph.org/educate/models/undergraduate-baccalaureate-cce-report/
